# Effects of different inhalant allergens on lung functions in adult patients with bronchial asthma

**DOI:** 10.1002/iid3.1118

**Published:** 2023-12-26

**Authors:** Jiangbo Liu, Xuejiao Qian, Xueyan Jie, Ping Jiang

**Affiliations:** ^1^ Department of Respiratory Medicine Tianjin First Central Hospital Tianjin China; ^2^ Department of Pulmonary and Critical Care Medicine Second Affiliated Hospital of Xi'an Jiaotong University Xi'an China

**Keywords:** adult patients, bronchial asthma, inhalant allergens, lung function

## Abstract

**Objective:**

To analyze the effects of different types of inhalant allergens on the lung functions of adult patients with bronchial asthma.

**Methods:**

This cross‐sectional study included a total of 47 adults diagnosed with bronchial asthma at the Respiratory Outpatient Department of Tianjin First Central Hospital. Patients were divided into non‐sensitized and sensitized groups based on the number of positive allergens detected and classified into four groups (the dust mite mixed group, animal dander mixed group, pollen‐mixed group, and mold mixed group) based on the type of positive allergen detected. They were tested for the serum concentration of allergen‐specific immunoglobulin E (sIgE) using a fluorescence immunoassay analyzer, and lung function was assessed using a pulmonary function testing machine. One‐way analysis of variance was used to compare normally distributed data, while the rank sum test was utilized for non‐normally distributed data.

**Results:**

There was no statistically significant difference in lung function indicators between these two groups (*p* > .05). There were statistically significant differences in forced expiratory volume in one second as a percentage of the predicted value (FEV_1_%pred) (*p* = .028), FEV_1_/forced vital capacity as a percentage of the predicted value; (FVC%pred) (*p* = .016), peak expiratory flow as a percentage of the predicted value (PEF%pred) (*p* = .001), forced expiratory flow at 50% of the predicted value of forced vital capacity (FEF50%pred) (*p* = .003), forced expiratory flow at 75% of the predicted value of forced vital capacity (FEF75%pred) (*p* = .023), and maximal midexpiratory flow (MM)EF75/25%pred (*p* = .002) among the four groups. The pollen‐mixed group had higher PEF%pred (pollen vs. animal dander, *p* = .067; pollen vs. dust‐mites, *p* = .008; pollen vs. molds, *p* = .001) and MMEF75/25%pred (pollen vs. animal dander, *p* = .048; pollen vs. dust‐mites, *p* = .003; pollen vs. molds, *p* = .001) than the other three groups. The pollen‐mixed group had higher FEF50%pred than the dust‐mites mixed group (*p* = .008) and molds‐mixed group (*p* = .001). The pollen‐mixed group had higher FEF75%pred (*p* = .005), FEV_1_%pred (*p* = .001), and FEV_1_/FVC%pred (*p* = .001) than the molds‐mixed group.

**Conclusion:**

Different inhalant allergens had different effects on lung functions in adults with asthma.

## 1 INTRODUCTION

Bronchial asthma, often referred to as asthma, is a complex and diverse disease characterized by chronic airway inflammation, involving a multitude of immune cells, airway structural cells, and inflammatory mediators.^1^ The progression of asthma is consistently linked to exposure to various inhalant allergens.^2^ When airway epithelial cells encounter these allergens, they release inflammatory substances, leading to smooth muscle cell hyperresponsiveness, excessive contractility, and remodeling of the airways.^3^ This cascade of events results in airflow limitation and a decline in lung function for the affected individual.^4^ Mold sensitization is associated with reduced lung function and increased airway hyperresponsiveness in asthmatics, especially in children with airflow limitation.^5^, ^6^ Moreover, allergens trigger asthma, resulting in persistent symptoms and a subsequent decline in lung function, which is associated with the severity of asthma.^7^ However, few studies directly compared the influence of various inhalant allergens on lung function in adult asthma patients. In this study, we aimed to categorize asthma patients based on their sensitization to different inhalant allergens and investigate the disparities in lung function among asthma patients who were sensitized to different inhalant allergens. Our findings may provide further insights into the effects of distinct inhalant allergens on airway damage in individuals with asthma.

## 2 MATERIALS AND METHODS

### 2.1 Clinical data

In this study, we included a total of 47 adults who were diagnosed with bronchial asthma at the Respiratory Outpatient Department of Tianjin First Central Hospital. Inclusion criteria: ① Patients were diagnosed with bronchial asthma in accordance with the *Guidelines for the Prevention and Treatment of Bronchial Asthma* (2020 Edition), recommended by the Bronchial Asthma Division of the Chinese Thoracic Society.^8^ The diagnostic criteria were as follows: 1. Clinical symptoms and signs: (1) Recurrent wheezing, shortness of breath, with or without chest tightness or cough, often worsened at night and in the early morning, frequently associated with exposure to allergens, cold air, physical, chemical irritants, upper respiratory infections, and exercise, among others; (2) During attacks and in some cases of uncontrolled chronic persistent asthma, scattered or diffuse wheezing can be heard in both lungs, with prolonged expiration; (3) The above symptoms and signs can be alleviated through treatment or spontaneously resolved. 2. Examination of airflow limitation: (1) Positive response to a bronchodilator. (2) Positive response to bronchial provocation. (3) Peak expiratory flow (PEF) daily diurnal variation rate > 10%, or PEF weekly variation rate > 20%. ② Patients aged ≥ 18 years. ③ Patients who were not in an acute asthmatic phase. Exclusion criteria: ① Patients who suffered from refractory asthma. ② Patients who had a respiratory tract infection in the past 4 weeks. ③ Patients with systemic or local use of hormones or anti‐allergic drugs in the past 3 months, as well as patients who were orally administered leukotriene receptor antagonists or bronchodilators in the past 2 weeks. ④ Asthma patients with additional respiratory diseases, such as chronic obstructive pulmonary disease, pneumonia, bronchiectasis, and so on, or other severe systemic diseases ⑤ Patients who were identified as unsuitable to participate in our trial procedures based on their lung function examination.

### 2.2 Methods

#### 2.2.1 Lung function examination

All participants underwent examinations for lung function under the professional guidance of physicians. The investigation data were collected and analyzed using a Jaeger Masterscreen Pulmonary Function Testing machine (Germany) in accordance with the Guidelines of the American Thoracic Society and the European Respiratory Society.^9^

#### 2.2.2 Detection of inhalant allergens through the serum sIgE test

A total of 2 mL of venous blood was collected from each participant. The blood samples were then subjected to centrifugation to separate the serum. The levels of immunoglobulin E (sIgE) and allergens were determined using the ImmunoCAP 250 automatic fluorescence immunoassay analyzer. The results were categorized as follows: sIgE levels < 0.35 kU/L were considered negative, while levels ≥ 0.35 kU/L were considered positive. Based on the results of tests, participants were divided into four groups: (1) Dust‐mites mixed group (house dust/house dust mite/powder dust mite, household dust mite/German cockroach); (2) animal dander mixed group (cat hair/dog hair/horsehair/cow hair); (3) pollens mixed group (ragweed/mugwort/French marigold/dandelion/Canadian goldenrod); and (4) molds‐mixed group (*Penicillium notatum*/*Cladosporium herbarum*/*Aspergillus fumigatus*/*Candida albicans*/*Alternaria*/*Helminthosporium*).

### 2.3 Statistical processing

We used SPSS 24.0 statistical software for statistical analysis of the data. All data in this study were tested for normality. Normally distributed measurement data were expressed as mean ± standard deviation, and non‐normally distributed measurement data were expressed as median (M) and interquartile range Q (P_75_–P_25_). For comparisons between multiple groups, we used one‐way analysis of variance for normally distributed data and the rank sum test for non‐normally distributed data. *p* < .05, or comparisons among multiple groups using *p* < .05 × 2/[*k* (*k* − 1)] indicated a statistically significant difference (where *k* represented the number of groups).

## 3 RESULTS

### 3.1 Analysis of clinical data

There were 33 participants in the sensitized group and 14 in the nonsensitized group. Among those in the sensitized group, 21 had a single allergen, including dust mites (*n* = 5), fungi (*n* = 5), and pollens (*n* = 11), and 12 had mixed allergens, including dust mites + animal hair (*n* = 6), dust mites + animal hair + fungi (*n* = 2), dust mites + animal hair + pollens (*n* = 1), dust mites + animal hair + fungi + pollens (*n* = 1), dust mites + pollens (*n* = 1), and fungi + pollens (*n* = 1).

There was no statistically significant difference in age and gender between the non‐sensitized and sensitized groups, as well as between the dust mites mixed, animal dander mixed, pollens mixed, and molds‐mixed groups (*p* > .05) (Tables 1 and 2).

**Table 1:** Comparison of age distribution and gender structure between the nonsensitized and sensitized groups.

Variables	Nonsensitized group (*n* = 14)	Sensitized group (*n* = 33)	*p*
Age (year)	44.64 ± 13.30	37.30 ± 14.31	.108
Gender (male/female)	6/8	16/17	.724

**Table 2:** Comparison of age distribution and gender structure among different inhalant allergen groups.

Variables	Dust‐mites mixed group (*n* = 16)	Animal dander mixed group (*n* = 10)	Pollens mixed group (*n* = 15)	Molds‐mixed group (*n* = 9)	*p*
Age (year)	33.88 ± 12.03	30.80 ± 11.21	33.00 ± 9.13	45.67 ± 21.00	.364
Gender (male/female)	8/8	5/5	7/8	6/3	.824

### 3.2 Comparison of lung function indicators between the nonsensitized and sensitized groups

There was no statistically significant difference between the non‐sensitized and sensitized groups in the following lung indicators: forced expiratory volume in one second as a percentage of the predicted value (FEV_1_%pred), FEV_1_/forced vital capacity as a percentage of the predicted value (FVC%pred), peak expiratory flow as a percentage of the predicted value (PEF%pred), forced expiratory flow at 50% of the predicted value of forced vital capacity (FEF50%pred), forced expiratory flow at 75% of the predicted value of forced vital capacity (FEF75%pred), maximum mid‐expiratory flow at 75/25% of the predicted value of MMEF (MMEF75/25%pred) (*p* > .05) (Table 3).

**Table 3:** Comparison of lung functions between the nonsensitized and sensitized asthma groups.

	Nonsensitized group (*n* = 14)	Sensitized group (*n* = 33)	*p*
PEF%pred	70.21 ± 20.32	77.04 ± 25.49	.379
MMEF75/25pred	43.49 ± 29.78	53.88 ± 31.31	.297
FEF50%pred	39.00 ± 59.10	60.80 ± 56.80	.450
FEF75%pred	30.25 ± 45.07	37.50 ± 50.45	.402
FEV_1_%pred	72.35 ± 35.28	82.80 ± 32.95	.500
FEV_1_/FVC%pred	66.47 ± 17.93	73.70 ± 27.02	.163

*Note*: Data are shown as mean ± standard deviation and compared by one‐way analysis of variance.

Abbreviations: FEF50%pred, forced expiratory flow at 50% of the predicted value of forced vital capacity; FEF75%pred, forced expiratory flow at 75% of the predicted value of forced vital capacity; FEV_1_%pred, forced expiratory volume in one second as a percentage of the predicted value; FVC%pred, forced vital capacity as a percentage of the predicted value; MMEF75/25%pred, maximum mid‐expiratory flow at 75/25% of the predicted value of MMEF; PEF%pred, peak expiratory flow as a percentage of the predicted value.

### 3.3 Comparison of lung function indicators in asthma patients with different inhalant allergens

**Figure 1:**
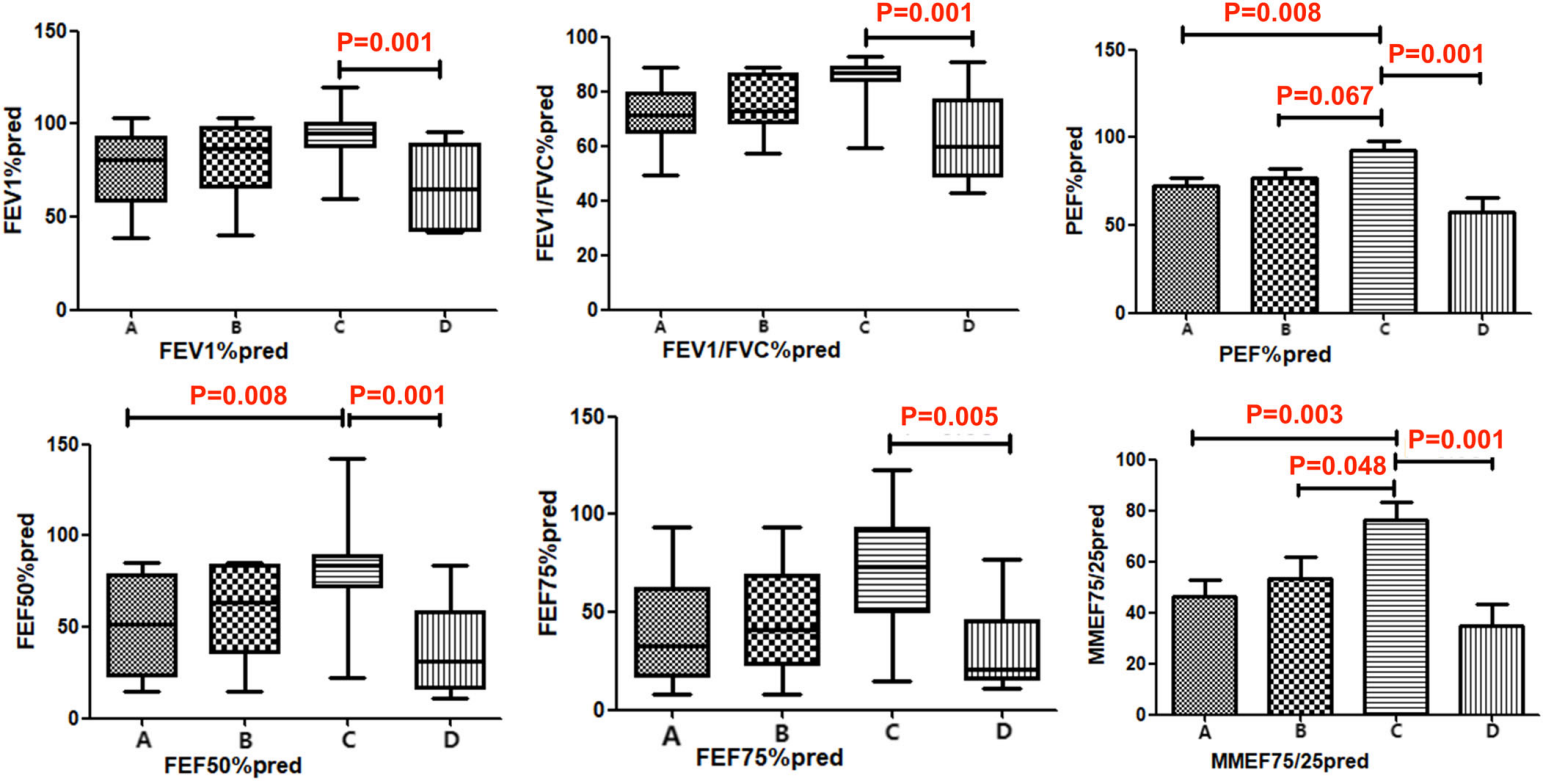
Comparison of lung functions among asthma patients with different inhalant allergens. A. Dust‐mites mixed group; B. Animal dander mixed group; C. Pollens mixed group. D. Molds‐mixed group. Data were expressed as median and interquartile range Q (P_75_–P_25_) and compared using the rank sum test.

There were statistically significant differences in FEV_1_%pred (*p* = .028), FEV_1_/FVC%pred (*p* = .016), PEF%pred (*p* = .001), FEF50%pred (*p* = .003), FEF75%pred (*p* = .023), and MMEF75/25%pred (*p* = .002) among the inhalant allergen groups of asthma patients, namely, the dust‐mites mixed, animal dander mixed, pollens mixed, and molds‐mixed groups. Based on further analysis through pairwise comparisons between these four groups, our findings were: The pollen‐mixed group had higher PEF%pred (pollen vs. animal dander, *p* = .067; pollen vs. dust‐mites, *p* = .008; pollen vs. molds, *p* = .001) and MMEF75/25%pred (pollen vs. animal dander, *p* = .048; Pollen vs. Dust‐mites, *p* = .003; pollen vs. molds, *p* = .001) than the other three groups. The pollen‐mixed group had higher FEF50%pred than the dust‐mites mixed (*p* = .008) and the molds‐mixed groups (*p* = .001). The pollen‐mixed group had higher FEF75%pred (*p* = .005), FEV_1_%pred (*p* = .001), and FEV_1_/FVC%pred (*p* = .001) than the molds‐mixed group (Figure 1).

## 4 DISCUSSION

In this study, we found that the non‐sensitized group had lower levels of all lung function indicators than the sensitized group, but the differences were not statistically significant. Miao et al.^10^ did not find significant differences in FEV_1_, FVC, and FEV_1_/FVC between children with non‐sensitized, single‐sensitized, and multi‐sensitized asthma. There was no statistically significant difference in FEV_1_%pred between adult patients with allergic asthma and patients with nonallergic asthma in the study by Wei et al.^11^ This suggests that there may be no difference in lung functions between patients with non‐sensitized and sensitized asthma.

Notably, despite the fact that no difference was observed in lung functions between patients with non‐sensitized and sensitized asthma, we found a trend that non‐sensitized patients had weaker lung functions than sensitized patients. Jang et al.^12^ proposed that patients with intractable asthma rarely had a clear allergen. According to epidemiological studies, exposure to various nonallergic irritants in the environment (ozone, respirable particulate matter, viruses or bacteria, cigarettes, occupational irritants, and so on) can induce neutrophilic inflammatory asthma and cause airway obstruction.^13^ The type of airway inflammation found in most steroid‐resistant and severely ill asthmatic patients is the neutrophil type.^14^

Other authors have proposed that adult patients with nonallergic asthma suffer more severe conditions than adult patients with allergic asthma, with a higher proportion of them experiencing severe and persistent conditions as well as irreversible airflow limitations.^10^ This suggests that it is important to strengthen monitoring of the condition and lung functions of non‐sensitized asthma patients to prevent their status from worsening into intractable asthma due to deficient lung functions.

As revealed by research, proteases contained in various types of inhalant allergens have pro‐inflammatory effects. They can damage the airway epithelium and cause epithelial cells to produce pro‐inflammatory factors, which can ultimately lead to airway remodeling.^15^ Pollen allergens are weaker in injuring airways than perennial allergens such as dust mites according to research by Boulet et al.^16^ and Liu et al.^17^ found that bronchial asthma patients, who were sensitized to weeds and pollens, had thinner airway walls and a higher FEF75/25%pred than those who were sensitized to household indoor dust mites.

The onset of asthma is closely related to inhalant allergens. Inhalant allergens can activate dendritic cells when they enter the airways, causing the dendritic cells to express costimulatory molecules and cytokines.^18^ This provides critical signals for the differentiation of naive CD4^+^ T cells into Th1, Th2, Th17, or Treg cells.^19^ Environmental allergens such as household indoor dust mites and cockroaches may be the main factors in the pathogenesis of asthma,^20^ and are associated with lung function impairment in patients. According to studies, in children with asthma, decreased levels of FEV_1_ and FEV_1_/FVC, which are indicators of lung function, are related to their exposures to indoor allergens.^21^ Gruzieva et al.^22^ suggested that exposure to pollens weakens the lung functions of children with asthma. Grant et al.^23^ found that the increases in FEV_1_ and FEF75/25 levels in patients with asthma were related to the decrease in their exposures to murine allergens.

Our findings in this study were that there were significant differences in both large and small airway functions between different inhalant allergen groups. Among them, the pollen‐mixed group had stronger lung functions than the other three groups, which is consistent with the above findings from other research works. Based on our analysis, this can be caused by the following aspects: (1) Diameters of pollen allergens. The diameters of pollen, dust mites, and fungi and spore allergens are 20⁠–60 μm, 1⁠–10 μm, and 3⁠–30 μm, respectively. How far an allergen particle can travel is determined by its size. The vast majority of large particles are removed by the nasal mucosa and upper respiratory tract, while only particles less than 3 μm in diameter can reach the bronchi and alveoli. Therefore, pollen particles, which are usually deposited in the upper airways, induce allergic rhinitis in most cases, whereas dust mites and fungi, which are small particles that can reach deep airways, induce asthma. When pollen particles shrink in diameter due to windy, humid weather, or on rainy days, as well as other factors, they enter the lower respiratory tract to induce bronchial asthma. (2) People only experience noticeable pollen allergens during specific seasons because pollens are seasonal allergens. In contrast, people can experience more severe airway inflammation and injuries if they are repeatedly exposed to perennial allergens like dust mites, animal dander, and molds.^24^, ^25^

Notably, in this study, we found that all the lung function indicators suggested that the molds‐mixed group had worse lung functions than the pollen‐mixed group, with the differences being statistically significant. Furthermore, all the lung function indicators suggested that the molds‐mixed group had the worst lung functions compared to the other three groups. Probable causes of these phenomena are as follows: According to previous research, in most cases, dust mites lead to eosinophilic inflammation^17^ and cell wall components of fungi (1,3‐β‐glucan) lead to neutrophilic inflammation.^13^ Neutrophilic inflammation is possibly linked to asthmas that are more intractable to control.^20^ Therefore, a possible reason for patients with mold‐allergic asthma having weaker lung functions than the other three groups is the type of airway inflammation. Therefore, in clinical practice, it is important to offer special attention to asthma patients who are sensitized to mold. They should be regularly examined for their lung functions, and treatments must be adjusted in time based on changes in their condition to prevent them from developing into irreversible airflow limitation due to worsening lung functions.

## 5 STRENGTHS AND LIMITATIONS

This being a cross‐sectional study, we were unable to do a dynamic analysis of the evolution and differences in lung functions between the non‐sensitized and sensitized groups, as well as between groups with different inhalant allergens, and this is an aspect that requires further investigation. This study is innovative in its detailed analysis of how lung function indicators were affected by varying dust mites, animal dander, molds, and pollens, which helped us conclude that there were differences in ventilation function and small airway function between different inhalant allergen groups. The severity of the asthmatic condition varied with the type of inhalant allergen. Individuals who were sensitized to pollens had better lung functions than those who were sensitized to other allergens. Patients in the molds‐mixed group had better lung functions, as evidenced by all lung function indicators, than the other three groups. In this study, we analyzed how different allergens could possibly affect lung functions, thus providing a reference for clinical personnel to explore the causes of intractable asthma. However, this study is limited due to its small sample size. We hope to conduct more in‐depth research based on larger sample sizes in the future.

## 6 CONCLUSION

In conclusion, we found that different inhalant allergens had varying effects on the lung functions of adult patients with asthma. Pollen allergens were less harmful to lung functions than other allergens, while mold allergens caused more severe harm to lung functions than other allergens. Therefore, in clinical practice, it is important to develop individualized treatment and follow‐up programs for asthma patients with different inhalant allergens, and greater attention should be focused on the changes in lung functions in adult patients with non‐sensitized asthma.

## AUTHOR CONTRIBUTIONS

**Jiangbo Liu**: Conceptualization; formal analysis; writing—original draft. **Xuejiao Qian**: Formal analysis; funding acquisition; project administration. **Xueyan Jie**: Data curation; formal analysis; writing—original draft.

## ETHICS STATEMENT

This study was approved by the Ethics Committee of Tianjin First Central Hospital (2019N134KY). This study was conducted in accordance with the declaration of Helsinki. Written informed consent was obtained from all participants.

## Data Availability

The data that support the findings of this study are available from the corresponding author upon reasonable request.
